# Exercise during pregnancy modulates infant cellular and whole‐body adiposity

**DOI:** 10.14814/phy2.70145

**Published:** 2024-12-01

**Authors:** Alex Claiborne, Filip Jevtovic, Donghai Zheng, Cody Strom, Breanna Wisseman, Samantha McDonald, Edward Newton, Steven Mouro, James DeVente, Joseph A. Houmard, Nicholas T. Broskey, Linda E. May

**Affiliations:** ^1^ Department of Kinesiology East Carolina University (ECU) Greenville North Carolina USA; ^2^ Human Performance Laboratory ECU Greenville North Carolina USA; ^3^ East Carolina Diabetes & Obesity Institute ECU Greenville North Carolina USA; ^4^ Department of Kinesiology and Sport University of Southern Indiana Evansville Indiana USA; ^5^ School of Kinesiology and Recreation Illinois State University Normal Illinois USA; ^6^ Department of Obstetrics & Gynecology East Carolina University Greenville North Carolina USA

**Keywords:** adipose, FIT‐V, mesenchymal stem cell, offspring, prenatal exercise

## Abstract

Non‐pharmaceutical interventions are needed to target the growing intergenerational cycle of obesity. We aimed to determine whether in utero exposure to different exercise doses during pregnancy directly reduces infant cellular and whole‐body adiposity. Pregnant women completed ~24 weeks of supervised exercise training; for standardization of exercise analysis (frequency, intensity, time, and volume‐FIT‐V), metrics were assessed from 16 to 36 weeks. Mesenchymal stem cells (MSC) collected from the umbilical cord at delivery underwent 21 days of adipogenic differentiation, then Oil Red O staining for lipid content. Infant body composition was measured at 1 month of age. ANCOVA and Pearson correlations determined the influence of prenatal exercise on infant adiposity. Exercise decreased infant MSC lipid content (*p* = 0.01) and body fat percentage (*p* = 0.009) irrespective of dose. Total exercise volume throughout pregnancy was negatively correlated with infant body fat % (*R* ^2^ = 0.31, *p* = 0.02) due to lower subscapular skinfolds (*R* ^2^ = 0.30, *p* = 0.02), while weekly exercise duration influenced adipogenic MSC lipid accumulation (*R* ^2^ = −0.23, *p* = 0.03) and BF% (*R* ^2^ = −0.15, *p* = 0.01). In utero exposure to exercise reduced cellular and whole‐body infant adiposity in a dose‐dependent manner.

## 1 INTRODUCTION

The excessive storage of adipose tissue in obesity contributes to the risk of cardiometabolic disease and mortality in adults and children (Kumanyika et al., 2008). Recent studies show that >30 million children under the age of 5 are overweight or obese (Childhood Overweight & Obesity, 2022; Vania, 2004), effectively perpetuating the current obesity epidemic. Increased lipid storage is present in mesenchymal stem cells (MSC) from offspring born to women with obesity (Boyle et al., 2016), thus suggesting that obesity begins to develop in fetuses in utero. Yet, when neonates are exposed to prenatal exercise in utero, body fat percentage is reduced compared to neonates of non‐exercising control (Claiborne, Wisseman, et al., 2023; Clapp & Capeless, 1990; Dieberger et al., 2023; McDonald et al., 2021; Menke et al., 2022; Norris et al., 2017). Specifically, data from previous investigations has shown that in utero exposure to aerobic exercise during pregnancy reduces offspring body fat percentage (Clapp, 1996; Clapp & Capeless, 1990; McDonald et al., 2021; Strom et al., 2022), therefore reducing the risk for childhood obesity (Bisson et al., 2017; Clapp & Capeless, 1990). Whether this reduction in overall adiposity is reflected by reduced lipid storage in infant adipocytes is yet to be tested.

Previous work has shown a robust response in cellular metabolism in offspring born to exercising pregnant women (Jevtovic et al., 2024; Jevtovic, Zheng, Houmard, Krassovskaia, et al., 2023). In particular, increased rates of fatty acid oxidation have been shown in undifferentiated MSC from exercise‐exposed offspring (Jevtovic, Zheng, Houmard, Krassovskaia, et al., 2023). This suggests that maternal exercise could result in increased fat utilization, and therefore less deposition of lipid droplets in adipogenically differentiated MSC which reflect adipocyte characteristics in offspring (Gyllenhammer et al., 2023). This is an important implication given previous evidence showing increased lipid accumulation in adipogenic MSC from offspring born to women with obesity (Boyle et al., 2016). In our larger parent trial, we have previously shown that exercise reduces infant adiposity markers (skin fold % body fat), and here we add analysis of lipid accumulation in offspring MSC. We therefore are interested in the direct comparison of prenatal exercise dose and lipid accumulation in adipogenic MSC, which has not yet been tested (Claiborne et al., 2024a, 2024b; May et al., 2010).

While the aforementioned studies have focused on recommended levels of aerobic exercise dose during pregnancy, investigation of exercise dose–response effects on infant adiposity have been lacking (Claiborne, Jevtovic, & May, 2023; May & Suminski, 2014). As highlighted in a recent review on exercise during pregnancy and infant outcomes (Claiborne, Jevtovic, & May, 2023), numerous investigations have shown reduced infant whole‐body adiposity but have not tested for dose‐dependent effects. Thus, it is critical to determine whether increased dose of exercise provides greater reduction in infant adiposity. These analyses can determine whether a higher exercise volume will provide additive benefits to normalizing infant adiposity. Recent work suggests that higher exercise volumes impart greater benefits for infant body composition (Bisson et al., 2017; Claiborne, Jevtovic, & May, 2023), and thus a higher dose may be necessary in overweight or obese maternal populations (Bisson et al., 2015).

This is the first study was conducted to assess the effect of prenatal exercise metrics (FITT‐V) among different types of exercise (aerobic, resistance, and combination) on infant cellular lipid accumulation in adipogenic mesenchymal stem cells as well as body composition (lean mass and body fat percentages) in exercise‐exposed offspring. We hypothesized that prenatal exercise would reduce cellular lipid accumulation and 1‐month infant body mass index through a decrease in body fat and increase in lean mass.

## 2 MATERIALS AND METHODS

### 2.1 Study participants

The current study was a secondary analysis of infant body composition at 1 month of age. In contrast with previously published reports on infant body composition response to maternal exercise (McDonald et al., 2021; Strom et al., 2022), data for the current study were compiled from two prospective, randomized control trials (RCTs) parsing the influence of prenatal exercise type on maternal and infant outcomes. The primary focus of this secondary analysis was to examine measures of offspring body composition and adipogenic MSC lipid accumulation in response to prenatal exercise. Specifically, we investigated the influence of prenatal exercise dose metrics: frequency, intensity, time, and volume. Women enrolled in these studies met the following criteria: clearance from a health care provider to participate in physical activity; between 18 and 40 years of age; prepregnancy body mass index (BMI; kg▪m^−2^) >18.5 kg▪m^−2^; singleton pregnancy; ≤16 weeks gestation; no current alcohol or tobacco use. Criteria for exclusion included smoking, known pre‐existing conditions (i.e., diabetes mellitus, hypertension, cardiovascular disease, and comorbidities, systemic lupus erythematosus), and/or medications known to affect fetal growth and well‐being.

### 2.2 Ethics statement

This study used records and umbilical cord mesenchymal stem cells (MSC) collected from participants enrolled in two RCTs investigating the influence of different maternal exercise types on infant outcomes (ClinicalTrials.gov Identifier: NCT03517293 and NCT03838146). Approval for these studies was obtained from the East Carolina University Institutional Review Board. Written informed consent was obtained from each participant upon enrollment. All experimental procedures were conducted at East Carolina University.

### 2.3 Pre‐intervention exercise testing and randomization

After study enrollment, participants completed a submaximal treadmill test to determine aerobic capacity and calculate target heart rate (THR) range for moderate‐intensity training. Peak oxygen consumption (VO_2_ peak) was estimated via the modified Balke protocol previously validated for pregnant women (Mottola, 2008). To minimize exposure risk after the start of the COVID‐19 pandemic, women recruited between March 2020 and October 2021 had THR zones for exercise based on their prepregnancy physical activity level and age (Mottola, 2008). THR zones for exercise components corresponded to maternal HR at 60%–80% of maximal oxygen consumption, that is, moderate intensity. After receiving clearance from the participant's obstetric provider, participants were randomly assigned into exercise (EX; aerobic AE, resistance RE, or combination AERE), or a non‐exercising control (CTRL) group.

### 2.4 Exercise intervention

All participants were supervised by trained exercise instructors in ECU facilities following a standard protocol. All sessions started at 16 weeks gestation and women were scheduled three times weekly until delivery (Birsner & Gyamfi‐Bannerman, 2015). All participant sessions included a 5‐min warm‐up, 50 min of their randomized group activity, and a 5‐min cooldown.

AE completed moderate intensity training on treadmills, ellipticals, recumbent bicycles, rowing, and/or stair‐stepping equipment. To maintain the appropriate HR zone, speed and grade were adjusted on the treadmill, and resistance and speed levels were adjusted on the elliptical and bicycle. RE completed sessions of two to three sets aiming for 12 repetitions of each exercise at ~60% of 1 repetition maximum (1‐RM) (Moyer et al., 2016). Exercises for RE were performed in a circuit with minimal rest (5–10 s) using seated machines (i.e., leg extension, leg curl, shoulder press, chest press, triceps extension, and latissimus dorsi pull‐down), dumbbells (i.e., biceps curls, lateral shoulder raises, and front shoulder raises), resistance bands, exercise balls, benches, and/or mats. AERE performed half of the aerobic protocol and half of the resistance protocol exercises in five circuits, lasting 4.5–5 min each. Resistance exercises were performed aiming for 12 repetitions (same exercises and equipment as the RE group), while aerobic exercises were performed on the same equipment as the AE group.

To ensure proper intensity was achieved during exercise sessions, the Borg scale rating of perceived exertion (RPE 6‐20), and “talk test” were used (Webster & Aznar‐Laín, 2008). HR monitoring (Polar FS2C, Kempele, Finland) ensured appropriate target HR ranges were maintained; target HR zones validated for pregnant women were utilized (Mottola, 2008).

### 2.5 Prenatal exercise dose

Exercising women aimed to complete three sessions per week, for 50 min per session, at moderate intensity. To control for differences in duration of pregnancy and start of intervention, exercise dose was analyzed from 16 to 36 weeks gestation for all participants.

Frequency was calculated as the number of times the participant attended supervised exercise each week and is expressed as sessions per week. Intensity was calculated using the published compendium of physical activity (Ainsworth et al., 2000, 2011) for each specific exercise performed. The average weekly intensity (METs) were calculated via average of these METs through all of the exercise sessions. Average weekly time was determined by exercise duration, in minutes for each week, with average duration reported as minutes/week. Lastly, prenatal exercise volume was calculated by (1) multiplying average intensity (METs) by the average weekly duration for volume in MET▪min/wk. (weekly exercise volume), and (2) multiplying weekly MET▪min by the total number of weeks prenatal exercise was performed through pregnancy for total MET▪min (total pregnancy volume).

### 2.6 Maternal measurements

Maternal age, parity, prepregnancy weight and height, gestational diabetes mellitus status (yes or no), 1‐h glucose value during an oral glucose tolerance test (OGTT), length of gestation, mode of delivery, and breastfeeding status were abstracted from various sources including prescreening eligibility and postpartum questionnaires as well as maternal and neonatal electronic health records. At 16 and 36 weeks of gestation maternal height and weight were obtained using a stadiometer and calibrated medical grade scale, respectively. Maternal prepregnancy BMI was calculated as [weight (kg)]/[height (m) ^2^] (healthy BMI 18.5–24.9; overweight 25–29.9, obese ≥30).

### 2.7 MSC isolation

Isolation of human infant MSCs from umbilical cord explants was completed as previously described (Jevtovic, Lopez, Zheng, Cortright, et al., 2023; Jevtovic & May, 2022; Jevtovic, Zheng, Houmard, Kern, et al., 2023b, 2023c; Jevtovic, Zheng, Houmard, Krassovskaia, et al., 2023; Jevtovic, Zheng, Lopez, Kern, et al., 2023). The sample size for adipogenic differentiation was limited to availability of clinical data for comparison, (infant adiposity measures, birth outcomes, and complete maternal FITT‐V exercise metrics) in subjects with cryopreserved umbilical MSC samples. While there is some overlap of subjects in the current study with those in previous publications from our group, seven of the 24 current subjects therefore were not included in previous reports. The umbilical cord explants were incubated in mesenchymal growth media containing low glucose DMEM (Gibco laboratories, Grand Island, NY) supplemented with 10% MSC‐qualified fetal bovine serum (FBS), 1x Gentamicin Amphotericin (Life Technologies, Gaithersburg, MD), until cells reached 80% to 100% confluency, then they were cryopreserved in liquid nitrogen until thawed and re‐seeded for adipogenic induction.

### 2.8 Adipogenic induction

For the metabolic studies, MSCs were cultured in mesenchymal growth media in 12‐well plates or T‐75 flasks until 80%–90% confluency (Table S1). To reduce the potential loss of the infant phenotype, all experiments were performed in similar MSC passages 3–5. Adipogenic induction was initialized 2–5 days post‐confluence as previously done (Boyle et al., 2016). For induction of adipogenesis, adipogenic induction media (AIM) containing low glucose DMEM (Gibco laboratories, Grand Island, NY) supplemented with 5% heat‐inactivated fetal bovine serum (FBS), 0.1X Penicillin Streptomycin (Life Technologies, Gaithersburg, MD), 1 μM dexamethasone, 0.2 mM indomethacin, 0.5 mM 3‐isobutyl‐1‐methylxanthine, and 170 nM insulin was used. Adipogenesis was maintained with adipogenic maintenance media (AMM) containing low glucose DMEM (Gibco laboratories, Grand Island, NY) supplemented with 5% heat‐inactivated fetal bovine serum (FBS), 0.1X Penicillin Streptomycin (Life Technologies, Gaithersburg, MD), and 170 nM insulin. Adipogenesis was performed for 21 days with 3‐day cycles of AIM (3d) – AMM (3d) – AIM (3d) – AMM (3d) – AMM (3d) – AMM (3d) – AMM (3d). All experiments were performed on the 21st day of adipogenic induction.

### 2.9 Lipid storage

For the assessment of natural lipid content, cells were rinsed with Dulbecco's Phosphate Buffered Saline (DPBS), fixed with 4% paraformaldehyde, and stained using 0.2% Oil Red O stain (ORO) in propylene glycol (Boyle et al., 2016). Thereafter, cells were rinsed with 85% propylene glycol and deionized water. ORO stain was solubilized with isopropanol and transferred to a clean 96‐well plate and analyzed via spectrophotometry at 520 nm for the degree of ORO staining. To account for cell density, each well was then stained with 0.3% Janus Green which provided a general cell count on each well. After rinsing with deionized water, cellular Janus Green was solubilized with 0.5 M HCl; samples were analyzed with spectrophotometry at 595 nm for degree of Janus Green staining, that is, cell count. Cellular ORO stain was then normalized to cellular Janus Green stain to estimate the lipid content per cell. All staining was done with five technical replicates.

### 2.10 Infant measurements

Birth measurements (weight, length, ponderal index, BMI, abdominal, head and chest circumference, 1 and 5‐min Apgar scores) and infant sex were extracted from neonatal electronic health records. At 1 month of age, infant weight (kg), length (m), BMI (kg/m^2^), and body fat (%), were measured by trained staff in a designated pediatric clinic as previously performed (McDonald et al., 2021; Strom et al., 2022). Weight and length were measured using a standard, calibrated infant scale and horizontal stadiometer, respectively. Skinfold thickness was measured via standard Lange calipers (BETA Technologies, Burlington, VT, USA) at three designated anatomical sites on the right side of the infant's body: biceps, triceps, and subscapular. Values for these sites were summed to determine skinfold thickness.

The skinfold thickness data was then used to calculate the percent body fat (BF%) using the following equation by Slaughter et al. (1988): Body Fat % = (1.21 * ([triceps] + [subscapular])) – ((0.008) * ([triceps] + [subscapular]) * ([triceps] + [subscapular])) – 1.7.

### 2.11 Statistical analysis

To assess the effect of prenatal exercise on maternal and infant characteristics, independent samples *t*‐tests were run between exercise and control groups with an *α* of *p* = 0.05. As we saw similar responses for each exercise (AE, RE, AERE), we combined these groups to power our comparisons. For comparison of the relationship between maternal exercise FIT‐V metrics, data were categorized into (1) above or below 450 MET▪min/wk, (2) above or below average intensity 4 METs, and (3) above or below 130 min weekly exercise duration based on a natural split of data, and independent samples *t*‐tests were used to determine differences between exercise doses. Analysis of covariance (ANCOVA) was performed to test the effect of exercise while controlling for gestational age at birth and prepregnancy fitness level. Finally, Pearson product–moment correlations were run between maternal exercise volume throughout pregnancy and adiposity outcomes. All statistical analyses were completed using SPSS software (version 28.0.1.1, SPSS Inc. IBM Corp., Chicago, IL).

## 3 RESULTS

MSC adipogenesis was performed on 24 (18 exercise‐exposed, 6 control) samples from healthy neonates. Infant body composition, lean mass, skinfold, and body fat data were available at 1 month of age for 16 of these infants. Maternal characteristics were similar between exercise and control (Table 1). Women participated in exercise for an average of 17 weeks during pregnancy. In assessing exercise dose from 16 to 36 weeks for standardization, exercise volume ranged between 200 and750 MET▪min/wk, spread over 30–71 total sessions, and resulting in 2000–18,000 total MET▪minutes through pregnancy.

**TABLE 1:** Participant characteristics.

Maternal	CON (*n* = 6)	EX (*n* = 18)	*p*
Pre‐Pregnancy BMI kg▪m^−2^	28.6 ± 4.4	28.1 ± 6.0	0.52
Age *years*	29.1 ± 5.5	29.8 ± 4.2	0.18
% BIPOC	43	11	0.08
Gravida	2 (1, 3)	1 (1, 3)	0.47
Parity	0 (0, 2)	0 (0, 2)	0.40
VO_2_peak ml▪kg▪min^−1^	18.4 ± 2.4	22.5 ± 3.7	0.37

Infant	CON (*n* = 6)	EX (*n* = 18)	*p*
% Female	38	42	0.65
GA at Birth *weeks*	39.0 ± 0.5	39.8 ± 0.5	0.42
% C‐Section	50	26	0.16

*Note*: Data reported as mean ± SD. Maternal characteristics measured before commencement of exercise (12–16 weeks of gestation) or documented at birth (pregnancy complications). Infant characteristics measured at birth.

Abbreviations: BIPOC, Black or Indigenous People of Color; BMI, body mass index; CON, control; C‐Section, caesarean section; EX, exercise; GA, gestational age; kg, kilograms bodyweight; m, meters height; min, minutes; ml, milliliters.

**FIGURE 1:**
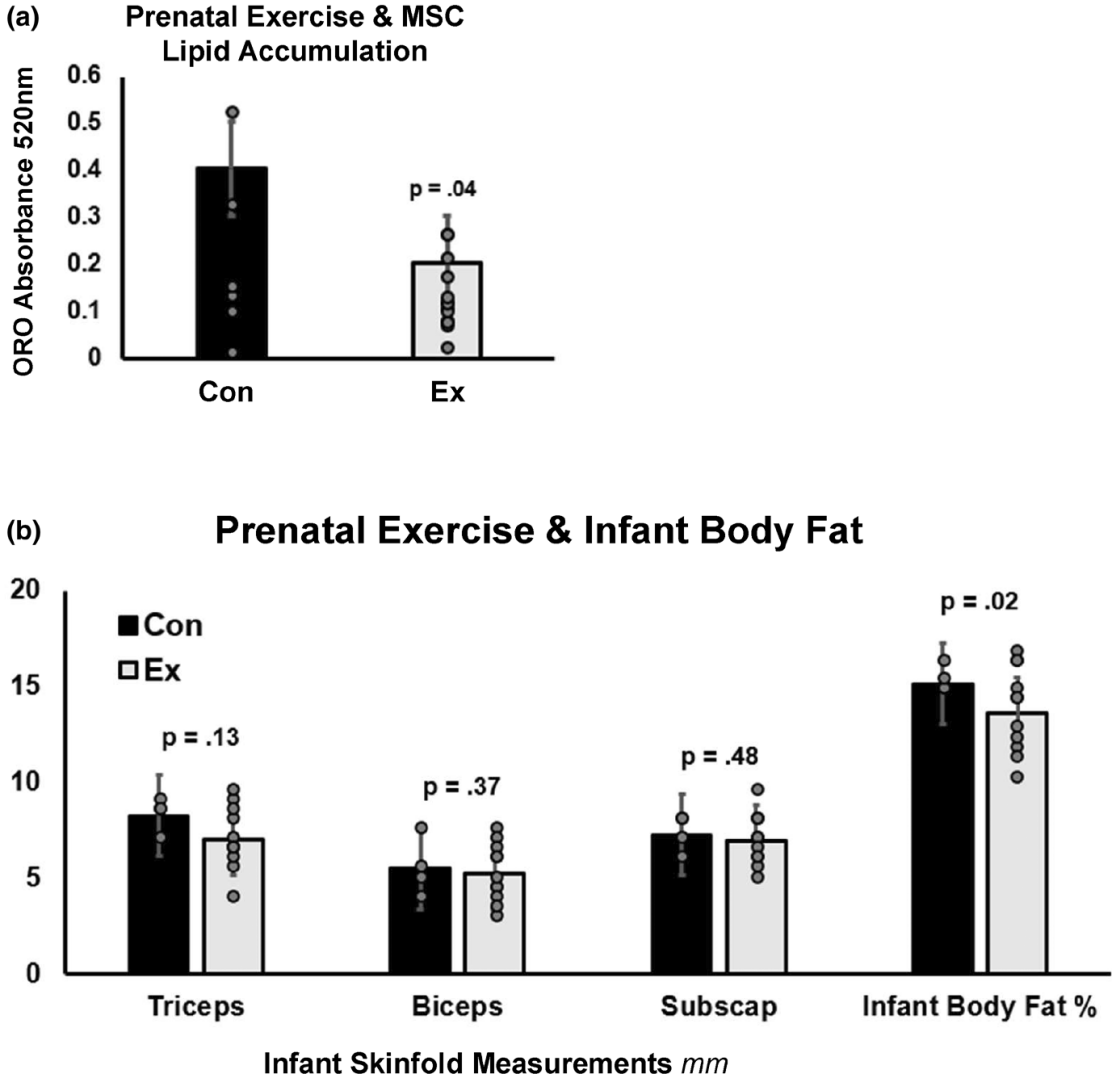
Prenatal exercise and infant adiposity. (a) Lipid accumulation measured by Oil Red O absorption at 520 nm in adipogenically differentiated umbilical MSCs between CON and EX groups. Individual skinfolds not different EX versus CON. (b) Infant skinfold measurements from 1 month of age between control (CON) and exercise (EX) groups. Infant body fat percentage (BF%) calculated from skinfold measurements taken in millimeters (mm).

There were no significant differences in infant birth characteristics between exercise‐exposed and control infants (Table 1). Exercise‐exposed infants displayed lower cellular lipid accumulation in adipogenic MSCs (Figure 1a; *p* = 0.01). In parallel, lower body fat percentage was seen in exercise‐exposed infants (Figure 1b; *p* = 0.009). Infant body mass index (BMI) at 1 month was lower (*p* = 0.05) with a nonsignificant 10% increase in infant lean mass % in exercise‐exposed (Table 1) offspring. After controlling for prepregnancy fitness level, a significant 50% reduction in cellular lipid storage with a clinically significant 1.6% decrease in 1‐month infant BF% was seen. Cellular lipid content was significantly correlated with infant BMI at 1 month (*R* = 0.63; *p* = 0.008), while no significant relationship was observed with BF% despite a high correlation coefficient (*R* = 0.35; *p* = 0.19). No significant differences were observed for adipogenic MSC lipid accumulation between exercise modes (ANOVA *p* = 0.78).

**FIGURE 2:**
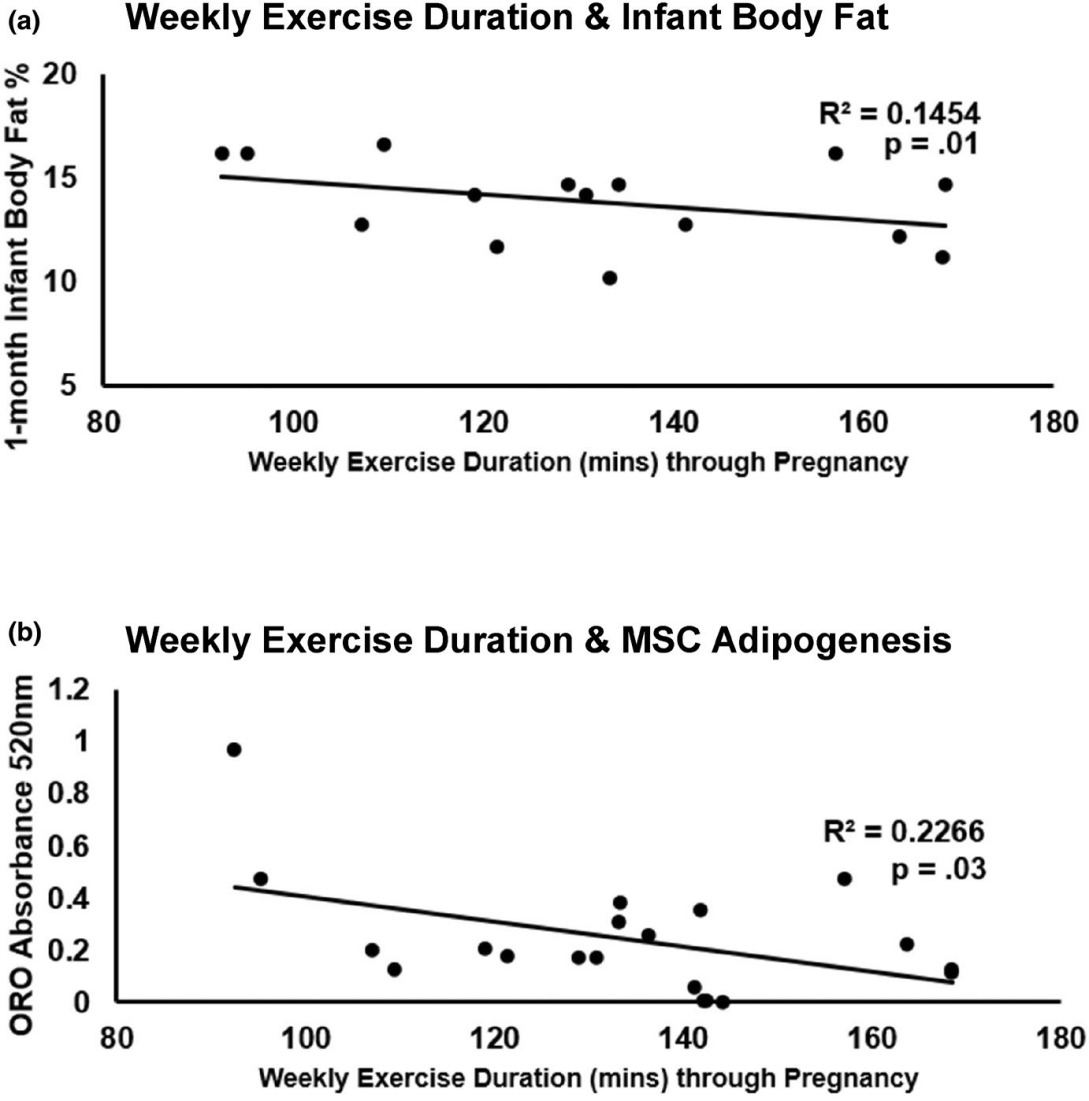
Weekly exercise duration and infant adiposity. Weekly exercise duration averaged from each week during pregnancy was significantly associated with infant subscapular skinfold thickness (a) and adipogenic MSC lipid accumulation (b).

**FIGURE 3:**
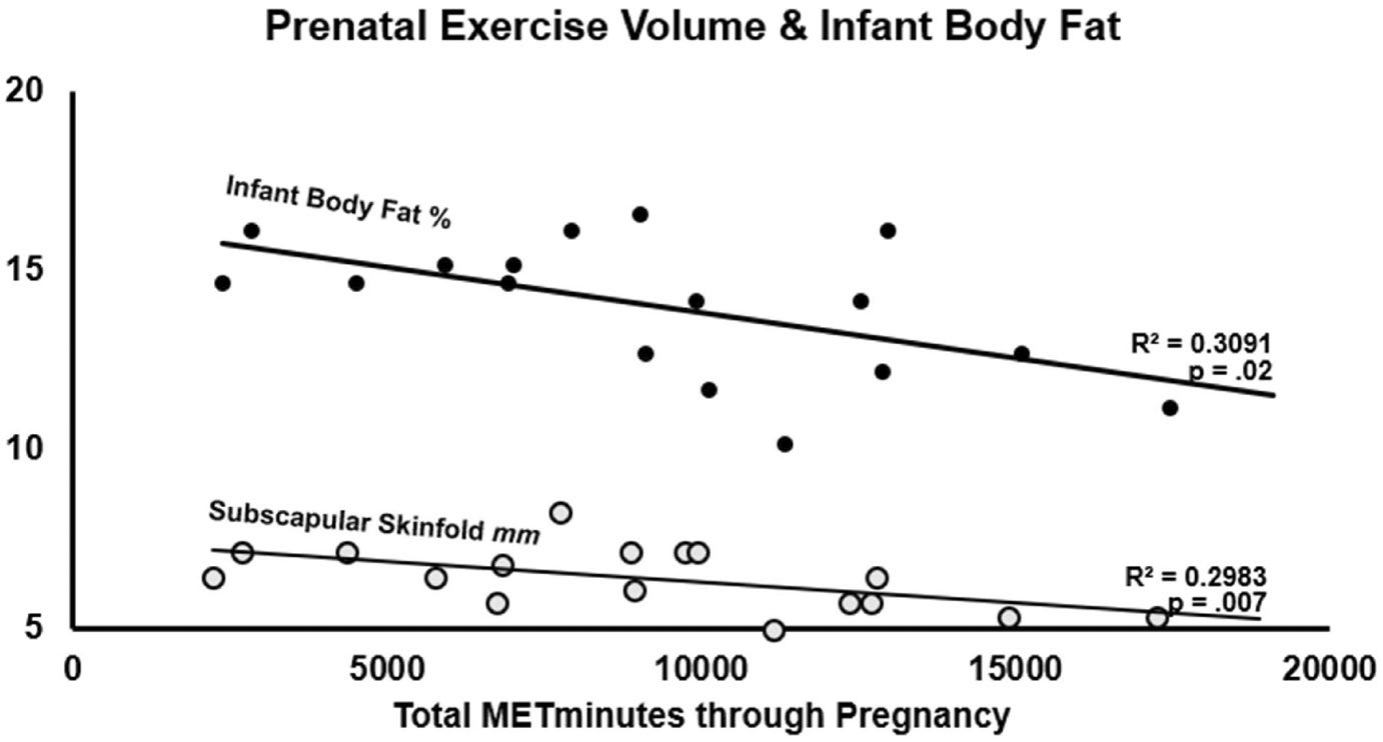
Total exercise volume and infant adiposity. Total MET▪min accumulated from exercise during pregnancy is associated with infant body fat at 1 month of age, due to the significant negative association with infant subscapular skinfold measurement.

When testing the influence of weekly exercise volume on infant adiposity, negative associations were found with infant cellular lipid storage (*p* = 0.07) and 1‐month BF% (*R* ^2^ = 0.31, *p* = 0.02). This association appeared to be due to significant negative associations of weekly exercise duration with infant lipid storage (Figure 2b; *R* ^2^ = 0.23, *p* = 0.03) and subscapular skinfold (Figure 2a; *R* ^2^ = 0.37, *p* = 0.02); while maintaining an average exercise intensity level >4 METs during exercise sessions led to a nonsignificant 3.5% increase in infant lean mass % (*p* = 0.35). For dose‐dependent relationships, total exercise volume throughout pregnancy was negatively correlated with infant BF% (Figure 3; *R* ^2^ = 0.31, *p* = 0.02). Additionally, total exercise volume was negatively correlated with subscapular skinfold measurement (*R* ^2^ = 0.30, *p* = 0.02), while no relationship was found for other skinfold sites.

## 4 DISCUSSION

The current study aimed to test the effect of prenatal exercise metrics on infant cellular lipid accumulation and whole‐body adiposity. We hypothesized that prenatal exercise would reduce infant adipogenic MSC lipid accumulation and 1‐month infant body mass index through a decrease in body fat and increase in lean mass. We found attenuated infant cellular lipid accumulation, which was mirrored by whole‐body adiposity. In parallel, we found prenatal exercise to reduce infant body mass index (BMI) and body fat percentage (BF%) in a dose‐dependent manner.

Our findings support the notion that cellular indices of infant adiposity such as lipid content mirror whole‐body adiposity reductions in exercise‐exposed offspring at birth. Others have utilized the umbilical MSC model with adipogenic differentiation to demonstrate the relationship between offspring cellular lipid content and in vivo infant BF% (Gyllenhammer et al., 2023), thus, demonstrating increased adiposity in infants born to women with obesity (Boyle et al., 2016). In the current study, we demonstrate that prenatal exercise decreases cellular lipid accumulation in infant adipogenic MSCs in a dose‐dependent manner, and that higher weekly and total exercise duration and volume enhance this reduction. These findings align with previous studies that show prenatal exercise contributes to decreased infant whole‐body adiposity (Jevtovic, Zheng, Houmard, Kern, et al., 2023b; McDonald et al., 2021; Strom et al., 2022). Thus, we believe that infants of women who meet the recommended exercise dose during pregnancy see greater benefits than infants of women who fall short of the recommended weekly exercise duration and volume (Birsner & Gyamfi‐Bannerman, 2015). This information provides a critical highlight to the importance of meeting the recommended dose at a minimum in women who desire to reduce offspring obesity risk.

The current findings support previous evidence that exercise performed during pregnancy is associated with reduced infant whole‐body adiposity (Clapp & Capeless, 1990; McDonald et al., 2021; Strom et al., 2022). Previous work has demonstrated the benefit of prenatal exercise on infant BF% from birth through 1 month of age (Clapp & Capeless, 1990; McDonald et al., 2020). The current study confirms that this effect is present when exercising throughout pregnancy, and that a specific reduction in subscapular adipose tissue could be responsible, which has been shown to be influenced by maternal metabolic flexibility and BMI (Tinius et al., 2021); thus, we suggest that prenatal exercise could reverse the negative influence of maternal obesity on offspring (Oken et al., 2007). Previous work has shown that increased exercise dose is associated with the reduction in infant adiposity (Claiborne, Jevtovic, & May, 2023; Clapp et al., 2002; Dieberger et al., 2023; Norris et al., 2017). Given the associated reduction of exercise duration and volume on infant body fat from the current and previous studies (Claiborne, Jevtovic, & May, 2023; Menke et al., 2022), research should continue to focus on the dose–response of offspring to maternal exercise exposure.

The current study has several strengths. Our findings are the first to assess the influence of supervised prenatal exercise metrics on infant adiposity at the cellular and whole‐body level. As complement to a strong randomized, controlled trial design, the current study utilizes supervised exercise across gestation at the recommended levels of exercise for pregnant women (Birsner & Gyamfi‐Bannerman, 2015). Our use of the umbilical MSC model with adipogenic differentiation provides an isolated investigation of prenatal exercise without external postnatal influence, while demonstrating a cellular link to lower adiposity in exercise‐exposed offspring. In addition to these strengths, we acknowledge some limitations. Firstly, our sample size was small, however, this still serves as the first observation of MSC adipogenic differentiation in an exercise during pregnancy model. Future works should test for more discrete differences in exercise offspring adipogenesis such as cell sizing, adipogenic propensity, and lipid transport. Secondly, our sample consisted of apparently healthy pregnancies, and the results might be limited in generalizability. We also did not measure real‐time energy expenditure during exercise sessions, thus, our estimates of METs are based on the correlation between target HR and exercise intensity measured at the initial treadmill test. Although there are limitations of skinfold measurement equations in children (Farmer, 1985) in estimating subcutaneous BF%, we selected the Slaughter equation which has been shown to highly (*R* = 0.90) correlate with body fat estimated by dual‐energy x‐ray absorptiometry (DXA) (Freedman et al., 2013); further, the use of this equation allows for comparisons with our previous reports. Future studies should confirm these findings with the use of DXA. Finally, since this was the first study assessing infant cellular lipid accumulation related to an exercise and pregnancy model, we were not able to perform a sample size justification, thus we recommend future studies address these questions with a larger sample.

## 5 CONCLUSION

While previous studies have shown neonatal adiposity is influenced by exercise during pregnancy, we now know that prenatal exercise is also associated with decreased cellular lipids in adipogenic MSCs. These findings further confirm the relationship of this model to in vivo changes in infants of exercising women. In parallel with infant cellular changes, we noted improved infant body composition at 1 month of age by attenuated body fat percentage, via higher duration and volume of exercise each week and throughout pregnancy further reduced infant adiposity. Total exercise volume throughout pregnancy was related to both reduced infant cellular lipid content and 1‐month body fat. Therefore, prenatal exercise exerts a dose–response effect which reduces infant adiposity at the cellular and whole‐body level and thus plays an important role in reducing the intergenerational cycle of obesity.

## AUTHOR CONTRIBUTIONS

Alex Claiborne performed experiments, analyzed data, interpreted results of experiments, prepared figures, drafted manuscript, edited and revised manuscript, approved final version of manuscript; Filip Jevtovic, Cody Strom, Breanna Wisseman, and Samantha McDonald performed experiments, interpreted results of experiments, edited and revised manuscript, approved final version of manuscript; Donghai Zheng, Joseph A Houmard, and Nicholas Broskey interpreted results of experiments, edited and revised manuscript, approved final version of manuscript; Edward Newton, Steven Mouro, and James deVente edited and revised manuscript, approved final version of manuscript; Linda E May conceived and designed research, interpreted results of experiments, edited and revised manuscript, approved final version of manuscript.

## FUNDING INFORMATION

American Heart Association grants #15GRNT24470029 (PI: May) and #18IPA34150006 (PI: May), Thrasher Research Foundation grant #02154 (PI: Claiborne), as well as internal funds provided by East Carolina University. Curation of funds only. Funding sources were not involved in study design or the collection, analysis, and interpretation of data.

## CONFLICT OF INTEREST STATEMENT

The authors report no conflict of interest.

## Supporting information


Table S1.

## Data Availability

Data generated/analyzed during the current study is available upon request from the corresponding/senior author.
