# Supplementary material for: Exercise during pregnancy modulates infant cellular and whole‐body adiposity
Source: Physiol Rep. 2024 Dec 1;12(23):e70145. doi: 10.14814/phy2.70145 (PMC11608828; doi:10.14814/phy2.70145)
Supplement: Supplementary file 1 — Table S1. [file PHY2-12-e70145-s001.docx]

SUPPLEMENTARY DATA

**Supplementary Table S1.** Supply and Reagent Reference

| **Supply** | **Manufacturer** | **Catalog Number** |
| --- | --- | --- |
| 6-well culture plates | VWR | 10062-892 |
| Low-glucose DMEM | Corning | 05124007 |
| MSC FBS | Gibco | 2453045RP |
| Dexamethasone | Thermo Fisher Scientific | AC230302500 |
| Indomethacin | Thermo Fisher Scientific | I7378-10G |
| 3-isobutyl methylxanthine | Thermo Fisher Scientific | I5879-1G |
| Insulin | Thermo Fisher Scientific | 15523-10MG |
| Oil Red O powder | Thermo Fisher Scientific | Z191058 |
| Janus Green powder | Thermo Fisher Scientific | 10222309 |
| 4% paraformaldehyde | Thermo Fisher Scientific | N16J500 |
| Propylene glycol | VWR Chemicals | 22I1456478 |
| Hydrochloric acid | Macron | H613-4S |
